# Application of mindfulness-based stress reduction plan in post-stroke patients with mild depression

**DOI:** 10.3389/fneur.2025.1629576

**Published:** 2025-09-30

**Authors:** Xiaofei Pang, Dong Qiu, Xiaowen He, Jiacheng Xu

**Affiliations:** Rehabilitation Medicine Center, Jinhua Hospital of TCM, Zhejiang, China

**Keywords:** mindfulness-based stress reduction, progressive resistance training, stroke, mild depression, depression

## Abstract

**Objective:**

To explore the therapeutic effect of mindfulness-based stress reduction in patients with post-stroke mild depression.

**Methods:**

A total of 80 patients with mild depression after stroke received by our hospital from January 2023 to December 2023 were selected as the study subjects. Randomly divide the patients into two groups: 40 in the control group and 40 in the combination group. The control group received conventional intervention therapy, while the combination group was treated with a mindfulness-based stress reduction plan combined with conventional intervention therapy. Patients in both groups were treated for 8 weeks. The general information of patients in the two groups was statistically analyzed. Self-rating Depression Scale (SDS), Hamilton Depression Rating Scale (HAMD-17), Mini Mental State Examination (MMSE), Montreal Cognitive Assessment (MoCA) scores and inflammatory factors (including TNF-α, hs-CRP, IL-6) expression levels were used to evaluate the efficacy of patients before and after treatment, and the total effective rate was counted for the two groups.

**Results:**

After treatment, compared with that before treatment, the HAMD-17 and SDS scores and inflammatory factor levels of patients in the control group and combination group were significantly decreased (all *p* < 0.05), while the MMSE and MoCA scores of patients in the control group and combination group were significantly increased (all *p* < 0.05). However, after treatment, the HAMD-17 and SDS scores and inflammatory factor levels of patients in the combination group were significantly lower than those in the control group (all *p* < 0.05). The scores of MMSE and MoCA were significantly higher than those in the control group (*p* < 0.05).

**Conclusion:**

In the treatment of stroke patients with mild depression, mindfulness-based stress reduction combined with conventional intervention therapy can improve the overall response rate compared with conventional intervention therapy alone and has a significant advantage in reducing depression.

## 1 Introduction

Stroke is a common cerebrovascular disease in middle-aged and elderly people, including cerebral hemorrhage and cerebral infarction (1, 2). The disease usually occurs suddenly with high mortality and disability rates, which seriously threatens the life safety and quality of life of patients. With the development of medical level, the mortality rate of stroke has been further controlled, but various complications caused by stroke still seriously affect the quality of life of patients. Depression is one of its common complications (3). Patients usually present with depression and unhappiness, chest tightness and discomfort, loss of interest or pleasure, and inattention (4, 5). Clinically, antidepressants are mostly used for treatment of patients with mild depression after stroke. However, the duration of drug treatment is long, and it is easy to relapse after treatment (6–8), so clinicians are looking for new non-drug methods to treat patients with mild depression. According to literature reports, mindfulness-based stress reduction can reduce the level of anxiety and depression in patients (9, 10), which has been widely used in patients with post-tumor depression and a low recurrence rate after improvement. This makes non-pharmacotherapy for depression a hot research field, but it is still relatively rare in the treatment of post-stroke depression. In this study, stroke patients with mild depression were treated by mindfulness-based stress reduction plan combined with conventional intervention therapy to provide a clinical basis for the promotion of non-pharmacotherapy in stroke patients with mild depression.

## 2 Data and methods

### 2.1 General data

This study was approved by the Medical Ethics Committee of the hospital. A total of 80 patients with mild depression after stroke admitted to our hospital from January 2023 to December 2023 were selected as the subjects. Inclusion criteria: (1) the degree of depression meets the diagnostic criteria for mild depression in ICD (international classification of diseases); (2) the diagnostic criteria for stroke established by the National Academic Conference on Cerebrovascular Diseases are met; 3 No antidepressant treatment was received before enrollment; 4 Hamilton Depression Rating Scale score is greater than or equal to 7 points and less than 17 points. Exclusion criteria: (1) previous history of depression; (2) accompanied by other serious diseases, such as cardiopulmonary dysfunction or tumor history; (3) accompanied by other mental disorder history; 4 Failure to complete the full course of treatment. They were divided into the control group and the combination group according to different intervention regimens, with 40 patients in each group. There was no significant difference in gender, age, education level, disease type and course of disease between the two groups (*p* > 0.05), but they were comparable (see Table 1). All patients gave informed consent for the study and signed an informed consent form.

**Table 1:** Comparison of general data between the two groups.

Group	*n*	Gender (male/female, *n*)	Age (years)	Education level (below middle school/above senior high school, *n*)	Disease type (cerebral hemorrhage/infarction, *n*)	Course of disease (d)
Control group	40	25/15	53.26 ± 11.60	27/13	13/27	15.42 ± 6.25
Combination group	40	24/16	54.20 ± 10.52	24/16	15/25	15.36 ± 6.13
*χ*^2^/t-value		0.0527	0.380	0.487	0.2120	0.043
*p*-value		0.819	0.705	0.485	0.639	0.966

### 2.2 Methods

Control group: Implement routine interventions by conducting a comprehensive assessment of the patient’s condition upon admission and understanding their level of disease awareness. Provide health education to both the patient and their family, explaining the causes, clinical manifestations, and other relevant knowledge. Focus on areas where the patient and family have limited understanding. Nursing staff should offer targeted psychological counseling to help patients develop a positive and optimistic mindset, encouraging active cooperation with treatment. Assess the patient’s previous dietary habits in detail, formulate an appropriate dietary plan based on their condition, and guide them in maintaining a balanced diet. Create a quiet and hygienic ward environment, and instruct patients to develop healthy sleep habits. Advise them to avoid consuming beverages such as coffee or strong tea before bedtime. Play soothing music before sleep to promote relaxation and improve sleep quality.

Combination group: Mindfulness-based stress reduction was added on the basis of the control group. Mindfulness-based stress reduction plan: (1) breathing meditation: in a quiet environment, the medical staff instructs the patient to take a supine position, place both hands on the patient’s abdomen, guide the patient to breathe calmly, feel the expansion of the abdomen during inhalation and the contraction of the abdomen during expiration, and pay attention to the patient’s breathing and emotional changes. The training time is once a day, 30 min/time. (2) Body scanning: The medical staff instruct the patient to take a supine position, and focus attention on the soles of feet, instep, calves, thighs, hips, back, shoulders, arms, head, face, etc. from bottom to top, while paying attention to the ups and downs of the abdomen, once/day, 15 min/ time. (3) Meditation: first feel the abdominal breathing for a period of time, then pay attention to the feeling of surrounding sounds, and pay attention to the pitch and timbre of sounds, once/day, 30 min/time.

### 2.3 Observation indicators

(1) HAMD-17, SDS, MMSE and MoCA scores and SDS score of patients before and after treatment (11): There are 20 items in total. The scale adopts a four-grade scoring method (1–4 points). The sum of the scores of each item is the total rough score, which is multiplied by 1.25 and taken as the standard score. The standard score of 53–62 is mild depression, 63–72 is moderate depression, and >72 is severe depression; HAMD-17 score (12): A total of 17 items were included, including the total score and 5 factor scores of physical anxiety, suicide, sleep disorder, despair and retardation. Each item was graded according to 0–4 levels. The higher the score, the more severe the depression degree. The higher the total score, the more severe the depression. <7 points indicate no depression, >17 points and ≤24 points indicate mild to moderate depression, and >24 points indicate severe depression; MMSE score (13): It includes 5 items, namely orientation, memory, attention, calculation, recall and language. After integration, the scores of each item are divided into normal (≥27 points), mild cognitive impairment (24–26 points) and moderate to severe cognitive impairment (≤23 points) according to the cognitive status of the subjects; MoCA score (14): It includes 8 items, namely, visual space and executive function, naming, memory, attention, language, abstraction, delayed recall and orientation. The total score is the sum of the scores of each cognitive domain, ranging from 0 to 33 points. If the education years are ≤12 years, 1 point will be added. The higher the score, the better the cognitive function. A score ≥26 indicates no cognitive impairment. (2) Gender, age, education level, disease type and course of disease of patients; (3) Clinical effect after treatment: Markedly effective if the HAMD-17 score of patients is below the depression level, effective response was defined as a ≥ 40% reduction in HAMD-17 score, and ineffective if the above criteria are not met after treatment. Total effective rate = (significantly effective + effective)/total number of cases × 100%; (4) Inflammatory factors: 3 mL of fasting venous blood was collected from patients in the morning, centrifuged for 10 min at a speed of 2,500 r/min, and serum was collected. Tumor necrosis factor-*α* (TNF-α), hypersensitive C-reactive protein (HSRP) hs-CRP and interleukin-6 (IL-6).

### 2.4 Statistical processing

SPSS21.0 statistical software was used to analyze and process the data. Measurement data were expressed as “ $\bar{x}$ ± *s*”, and *t*-test was used for comparison between groups; enumeration data were expressed as cases or %, and *χ*^2^ test was used for comparison between two groups. *p* < 0.05 was considered statistically significant.

## 3 Results

### 3.1 Comparison of clinical effects between the two groups

The total effective rate of the combination group was significantly higher than that of the control group (*χ*^2^ = 6.13, *p* < 0.05) (see Table 2).

**Table 2:** Comparison of clinical effects between the two groups.

Group	*n*	Significantly effective	Effective	Ineffective	Overall response rate (*n*, %)
Control group	40	12	20	8	32 (80.00)
Combination group	40	17	22	1	39 (97.5)
*χ* ^2^	–	–	–	–	6.13
*p-*value	–	–	–	–	0.01

### 3.2 Comparison of general data between the two groups

The general data of the participants who completed the whole process in the two groups were counted, including gender, age, education level, disease type and duration of disease course. The results showed that there was no statistically significant difference in general data between the two groups (all *p* > 0.05) (see Table 1).

### 3.3 Comparison of depression scores before and after treatment between the two groups

Before treatment, there was no significant difference in depression scores (including HAMD-17 and SDS scores) between the control group and the combination group (all *p* > 0.05); After treatment, the scores of HAMD and SDS in control group and combination group were significantly lower than those before treatment (all *p* < 0.05); After treatment, the HAMD-17 and SDS scores of patients in the combination group were significantly lower than those in the control group (all *p* < 0.05) (see Table 3).

**Table 3:** Comparison of SDS and HAMD-17 scores before and after treatment.

Group	*n*	SDS score		HAMD score	
		Before treatment	Post-treatment	Before treatment	Post-treatment
Control group	40	59.42 ± 2.93	45.62 ± 2.43*	14.65 ± 2.17	8.65 ± 3.12^*^
Combination group	40	61.25 ± 3.04	34.15 ± 2.12*^△^	14.12 ± 2.35	5.14 ± 2.10*^△^

*Refers to *p* < 0.05 compared to Before treatment; ^△^Refers to *p* < 0.05 compared to Control group.

### 3.4 Comparison of MoCA and MMSE scores between the two groups

**Figure 1:**
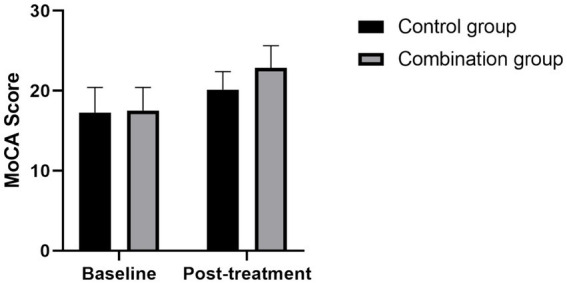
Comparison of MoCA scores between the two groups before and after treatment Compared with the control group, the MoCA scores in the combined group was significantly higher after treatment (*p* < 0.05).

**Figure 2:**
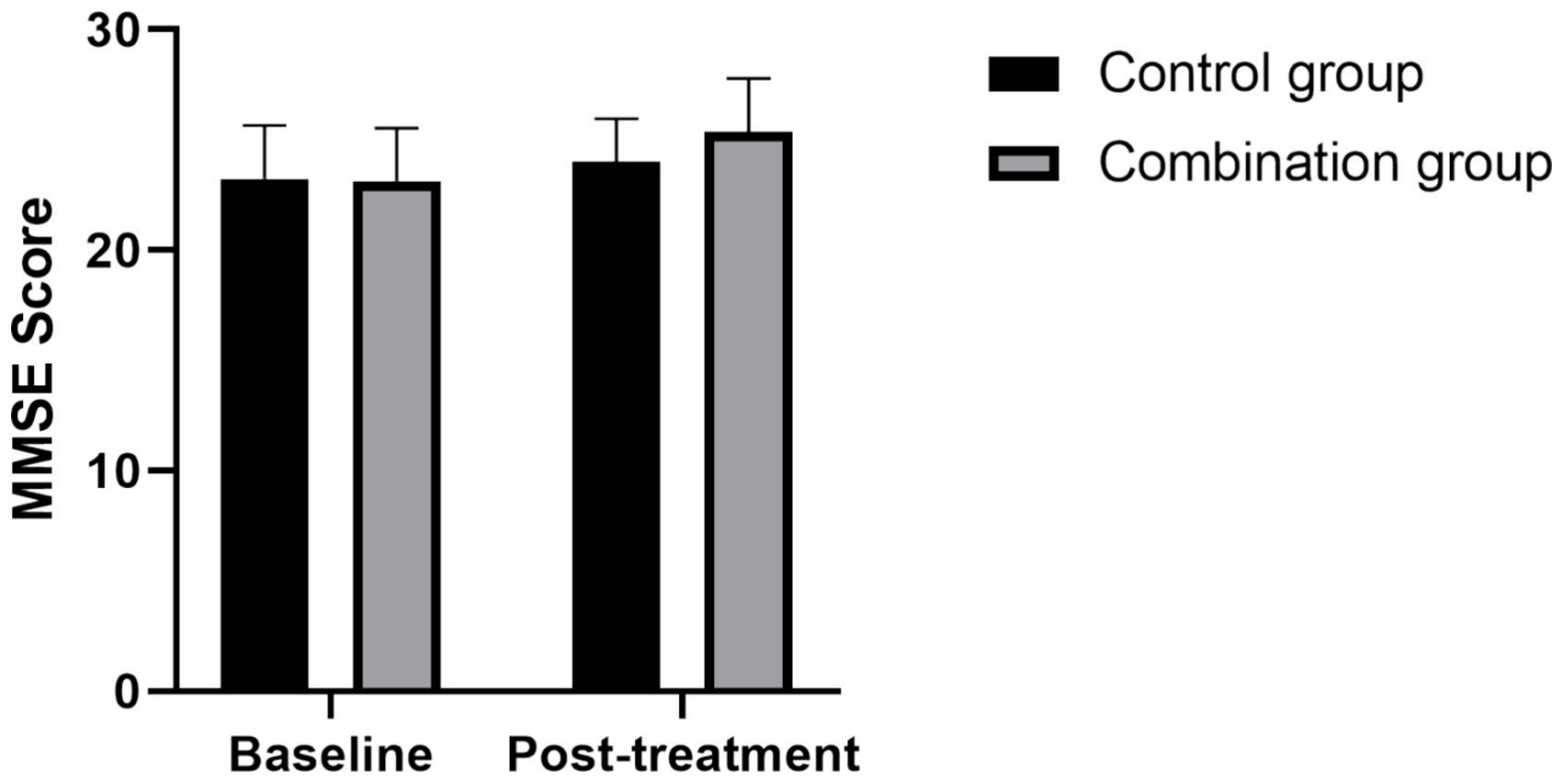
Comparison of MMSE scores between the two groups before and after treatment Compared with the control group, the MMSE scores in the combined group was significantly higher after treatment (*p* < 0.05).

Before treatment, there was no statistically significant difference in MoCA and MMSE scores between the control group and the combination group (all *p* > 0.05); after treatment, compared with that before treatment, the MoCA and MMSE scores of patients in the control group and the combination group were significantly increased (all *p* < 0.05); after treatment, the MoCA and MMSE scores of patients in the combination group were significantly higher than those in the control group (all *p* < 0.05) (see Figures 1, 2).

### 3.5 Comparison of inflammatory factors between the two groups

Before treatment, there was no significant difference in the levels of inflammatory factors (including TNF-α, hs-CRP and IL-6) between the control group and the combination group (all *p* > 0.05); After treatment, the inflammatory factors of control group and combination group decreased significantly compared with those before treatment (all *p* < 0.05); After treatment, the inflammatory factors of patients in the combination group were significantly lower than those in the control group (all *p* < 0.05) (see Table 4).

**Table 4:** Comparison of inflammatory factor levels.

Group	*n*	TNF-α (ng/L)		hs-CRP (mg/L)		IL-6 (ng/L)	
		Before treatment	Post-treatment	Before treatment	Post-treatment	Before treatment	Post-treatment
Control group	40	29.43 ± 4.51	23.10 ± 4.07	6.58 ± 1.07	5.43 ± 0.91	26.29 ± 4.13	20.46 ± 2.89
Combination group	40	28.69 ± 5.25	17.85 ± 3.42	6.70 ± 1.12	4.35 ± 0.72	25.49 ± 3.22	16.54 ± 3.24
*t*-value		0.676	6.246	0.490	5.886	0.966	5.710
*p*-value		0.501	<0.001	0.626	<0.001	0.337	<0.001

## 4 Discussion

Stroke is a common cerebrovascular disease in the elderly, which usually has rapid onset and high mortality. Patients with stroke are often accompanied by a significant decrease in motor ability, which not only significantly reduces the quality of life of patients but may also have a serious impact on their mental health. Post-stroke depression (PSD) is a common complication (15, 16). It is an affective disorder that usually manifests as depressed mood, cognitive decline and decreased interest. In severe cases, it may even lead to suicidal behavior (17). There is a large difference in the incidence of PSD in relevant reports, which may be related to different selection and assessment methods by researchers. At present, the standard treatment for depression includes drug therapy and psychotherapy, but there is still uncertainty about the efficacy of these treatments in stroke patients (18–20). Although drug therapy is effective quickly, it also faces the problem of high recurrence rate (21). Studies have shown that psychological intervention can effectively reduce the recurrence rate of depression (22, 23). Therefore, there is an urgent clinical need for a safe and effective non-pharmacological treatment to deal with PSD. Conventional psychological interventions have previously achieved good therapeutic effects, but their efficacy remains limited and ineffective for some patients. This study aimed to explore the possibility of treating PSD with a combined intervention of mindfulness-based stress reduction and conventional intervention therapy. Mindfulness-based stress reduction is a technique to reduce stress by improving the individual’s awareness of the current moment. This combination method may provide a new scientific basis and practical guidance for non-pharmacological treatment of post-stroke depression. Through this study, we hope to provide more options for clinical practice that will help improve the quality of life and overall well-being of patients with PSD.

Mindfulness-based stress reduction (MBSR) was pioneered by Dr. Jon Kabat-Zinn at the University of Massachusetts Medical Center in 1979 (24). This therapy is designed to help patients with various medical conditions relieve symptoms such as pain, anxiety and depression through the use of mindfulness techniques. The concept of mindfulness, derived from the Buddhist and Yogic traditions, emphasizes a conscious and non-judgmental focus on immediate experiences, including aspects of one’s feelings, thoughts, and moods. Mindfulness-based stress reduction is a systematic psychological intervention method, which usually includes exercises such as sitting meditation, body scanning and thinking (25–27). The standard treatment cycle is 8 weeks. Initially, Mindfulness-based stress reduction was used in clinical practice as an adjuvant therapy. With the gradual emergence of its efficacy, its application has gradually expanded to chronic physical diseases, mental illness rehabilitation and other fields (28). In recent years, mindfulness-based stress reduction has gained wide attention and acceptance in the treatment of post-tumor mood disorders (29, 30), showing good clinical results. However, for patients with mild depression after stroke, there are still relatively few studies on the application and effect of mindfulness-based stress reduction, which is also a direction that this study focuses on exploring. This study conducted an intervention experiment on stroke patients with mild depression. Patients were divided into two groups according to different intervention methods: The control group received conventional intervention therapy, while the combination group was treated with a mindfulness-based stress reduction plan combined with conventional intervention therapy. The study results showed that the overall clinical response rate of patients in the combination group was significantly higher than that in the control group (*χ*^2^ = 6.13, *p* < 0.05). In addition, compared with the control group, the combination group showed significantly lower scores in Hamilton Depression Rating Scale (HAMD-17), Self-rating Depression Scale (SDS) and levels of inflammatory factors (including TNF-α, hs-CRP, IL-6) (all *p* < 0.05), while also showed significant advantages in Mini Mental State Examination (MMSE) and Montreal Cognitive Assessment (MoCA) scores (all *p* < 0.05).

In conclusion, both conventional intervention therapy and mindfulness-based stress reduction are safe and effective in the treatment of mild depression after stroke. However, mindfulness-based stress reduction combined with conventional intervention therapy can significantly improve the total clinical response rate of patients and significantly reduce their depressive symptoms, which provides an important reference for promoting non-pharmacotherapy in the treatment of mild depression after stroke.

## Data Availability

The raw data supporting the conclusions of this article will be made available by the authors, without undue reservation.
